# Polybrominated Diphenyl Ethers (PBDEs) in Breast Milk and Neuropsychological Development in Infants

**DOI:** 10.1289/ehp.1205266

**Published:** 2012-09-25

**Authors:** Mireia Gascon, Marta Fort, David Martínez, Anne-Elie Carsin, Joan Forns, Joan O. Grimalt, Loreto Santa Marina, Nerea Lertxundi, Jordi Sunyer, Martine Vrijheid

**Affiliations:** 1 Centre for Research in Environmental Epidemiology (CREAL), Barcelona, Catalonia, Spain; 2 Hospital del Mar Research Institute (IMIM), Barcelona, Catalonia, Spain; 3 Spanish Consortium for Research on Epidemiology and Public Health (CIBERESP), Barcelona, Catalonia, Spain; 4 Department of Environmental Chemistry, Institute of Environmental Assessment and Water Research (IDAEA-CSIC), Barcelona, Catalonia, Spain; 5 Departamento de Sanidad Gobierno Vasco, Subdirección de Salud Pública de Gipuzkoa, Donostia-San Sebastián, Spain; 6 Biodonostia, Instituto de Investigación Biomédica, Donostia-San Sebastián, Spain; 7 Department of Social Psychology and Behavioral Sciences Methodology, Faculty of Psychology, University of the Basque Country (UPV/EHU), Donostia-San Sebastián, Spain; 8 Universitat Pompeu Fabra (UPF), Barcelona, Catalonia, Spain

**Keywords:** children, environmental, neurodevelopment, persistent organic pollutants (POPs), polybrominated diphenyl ethers (PBDEs)

## Abstract

Background: There is increasing interest in the potential effects of polybrominated diphenyl ethers (PBDEs) on children’s neuropsychological development, but only a few small studies have evaluated such effects.

Objectives: Our goal was to examine the association between PBDE concentrations in colostrum and infant neuropsychological development and to assess the influence of other persistent organic pollutants (POPs) on such association.

Methods: We measured concentrations of PBDEs and other POPs in colostrum samples of 290 women recruited in a Spanish birth cohort. We tested children for mental and psychomotor development with the Bayley Scales of Infant Development at 12–18 months of age. We analyzed the sum of the seven most common PBDE congeners (BDEs 47, 99, 100, 153, 154, 183, 209) and each congener separately.

Results: Increasing Σ_7_PBDEs concentrations showed an association of borderline statistical significance with decreasing mental development scores (β per log ng/g lipid = –2.25; 95% CI: –4.75, 0.26). BDE-209, the congener present in highest concentrations, appeared to be the main congener responsible for this association (β = –2.40, 95% CI: –4.79, –0.01). There was little evidence for an association with psychomotor development. After adjustment for other POPs, the BDE-209 association with mental development score became slightly weaker (β = –2.10, 95% CI: –4.66, 0.46).

Conclusions: Our findings suggest an association between increasing PBDE concentrations in colostrum and a worse infant mental development, particularly for BDE-209, but require confirmation in larger studies. The association, if causal, may be due to unmeasured BDE-209 metabolites, including OH-PBDEs (hydroxylated PBDEs), which are more toxic, more stable, and more likely to cross the placenta and to easily reach the brain than BDE-209.

Polybrominated diphenyl ethers (PBDEs) are flame retardant additives used in plastics, electronics, foams, and textiles, and they have the capacity to bioaccumulate for periods of time ranging from a few days to around 7 years depending on the congener (Hooper and McDonald 2000; Thuresson et al. 2006; Trudel et al. 2011). PBDEs are widely distributed around the world, but levels can differ greatly between regions and countries (Betts 2002; Fangstrom et al. 2008). Although both in the environment and in biological matrices, including human, levels of the so-called “old” persistent organic pollutants (POPs), such as polychlorinated biphenyls (PCBs) or hexachlorobenzene (HCB), have been decreasing in the past decades because they have been banned (Nickerson 2006), levels of PBDEs have increased (Fischer et al. 2006; Toms et al. 2009). Also, sources of exposure can differ between PBDEs and these other POPs; dietary intake is commonly accepted to be the main exposure route for many POPs, such as PCBs and persistent pesticides (Llop et al. 2010; Polder et al. 2010). However, although diet can also be a route of human exposure to PBDEs, other sources such as dust inhalation, ingestion, or dermal absorption may be more relevant sources of exposure than diet (Frederiksen et al. 2009; Johnson et al. 2010; Liberda et al. 2011; Sjödin et al. 2008; Trudel et al. 2011; Watkins et al. 2012). Despite different routes of exposure, POPs (mainly PCBs) and PBDEs are structurally and chemically similar, and animal studies have reported neurotoxic effects of both classes of compounds, although PCBs appear to be stronger neurotoxicants than PBDEs (Branchi et al. 2003; Fonnum and Mariussen 2009; He et al. 2009a). Because PCBs, dichlorodiphenyldichloroethylene (DDE), and HCB have been associated with deleterious effects on children’s cognitive function and behavior at different ages (Eskenazi et al. 2009; Ribas-Fito et al. 2001, 2007b), there is increasing interest in the potential effects of PBDEs on children’s neuro-psychological development. Also, recent *in vitro* and animal studies suggest synergistic effects of co-exposure to PCBs and PBDEs (He et al. 2009b, 2011; Pellacani et al. 2012).

So far only a few small epidemiological studies (numbers of participants ranging from 36 to 118) show inconsistent associations between prenatal PBDEs exposure and neuro-psychological development between 1 and 6 years of age (Chao et al. 2011; Gascon et al. 2011; Herbstman et al. 2010; Roze et al. 2009; Shy et al. 2011). One study (Gascon et al. 2011) also estimated the effects of postnatal exposure in children 4 years of age, finding higher PBDE concentrations to be associated with poor social competence and attention deficit symptoms. Most of these studies measured PBDE exposures in cord blood or maternal serum. However, in western European populations PBDE levels are low and often hard to detect in these biological matrices because of their low lipid content. Because breast milk (particularly colostrum, the first breast milk after giving birth) is rich in fat content, it may be a better matrix for measuring highly lipophilic compounds such as PBDEs (Antignac et al. 2009; Gomara et al. 2007), and thus may be more suitable than cord blood or maternal serum for assessing prenatal exposure to PBDEs.

The aims of the present study were to examine the association between PBDE concentrations in colostrum and infant neuro-psychological development, to assess the role of individual PBDE congeners on neuro-psychological development, and to assess whether the estimated effects of PBDEs are modified and/or enhanced by co-exposure to organochlorine compounds (OCs) such as DDE, HCB, and PCBs.

## Methods

*Study population.* This study is based on two Spanish regions (Gipuzkoa, Basque Country; and Sabadell, Catalonia) belonging to the INMA—INfancia y Medio Ambiente (Environment and Childhood)—Project in Spain (Guxens et al. 2012). These regions followed the same protocol and started recruiting pregnant women into the cohort between 2004 and 2008 (Sabadell *n* = 657, Gipuzkoa *n* = 638). Protocol details are described elsewhere (Guxens et al. 2012). In summary, pregnant women were recruited during the first trimester routine antenatal care visit in the main public hospital or health center of reference if they fulfilled the following inclusion criteria: age ≥ 16 years, intention to deliver in the city, singleton birth, no assisted conception, and no problems with communication. The study was conducted with the approval of the hospital ethics committees in the participating regions, and written informed consent was obtained from the -parents of all children.

From the 1,295 initially recruited -mother–children pairs, 62 were lost-to -follow-up due to miscarriages (*n* = 29), fetal death (*n* = 4), or voluntary withdrawal (*n* = 29). Of the 1,233 remaining children, 492 mothers provided colostrum samples, in which polyunsaturated fatty acids (PUFAs) were measured for a previous study (Guxens et al. 2011). After analyses of PUFAs, 348 samples remained and were available for measurement of PBDEs and other POPs. From these 348 children with information on exposure levels, 29 had no neuro-psychological testing, 18 were excluded because their test results were of uncertain quality due to less than optimal cooperation of the child, and 11 were excluded because they had a gestational age < 37 weeks. In total, 290 children, 88 from Gipuzkoa and 202 from Sabadell, were included in the analysis.

*Child neuropsychological assessment.* We assessed children’s cognitive and psychomotor development at around 14 months of age (range, 12–18 months) using the Bayley Scales of Infant Development (BSID) (Bayley 1977). The mental scale consists of 163 items that assess age-appropriate mental development, including performance abilities, memory, and early language skills. The psychomotor scale consists of 81 items assessing fine and gross motor development. All testing was done in the health care center by one of the four specially trained psychologists in the presence of the mother. Psychologists were not aware of any exposure information. To limit interobserver variability, we applied a strict protocol, including training sessions where interobserver differences were quantified. Three sets of interobserver-reliability tests were conducted for each rater during fieldwork, and resulting intraclass correlations were 0.90 for the BSID mental scale and 0.91 for the psychomotor scale. Cronbach’s alpha coefficient, a measure of internal consistency, was 0.78 for the mental scale and was 0.73 for the psychomotor scale. Scores were standardized for child’s age in days at test administration using a parametric method to estimate age-specific reference intervals. Specifically, parameters of the distribution were modeled as a fractional polynomial function of age and estimated by maximum likelihood, and residuals were normalized to a mean (± SD) of 100 ± 15 points to homogenize the scales and facilitate comparisons with other studies.

*Exposure assessment.* Colostrum samples were collected at the hospital during the first 48–96 hr postpartum by an experienced nurse using a breast pump, and were stored at –80ºC. We extracted OCs (DDE, HCB, and PCB congeners 28, 52, 101, 118, 138, 153, 180) and PBDEs (congeners 17, 28, 47, 66, 71, 85, 99, 100, 138, 153, 154, 183, 190, 209) following a liquid–liquid extraction protocol [described in detail in Supplemental Material, pp. 2–3 (http://dx.doi.org/10.1289/ehp.1205266)]. Limits of detection (LOD) and quantification (LOQ) were calculated from blanks (i.e., the SD times three LOD or five LOQ). Only those congeners detected in > 20% in colostrum samples were included in the total sum of seven PBDEs (Σ_7_PBDEs: 47, 99, 100, 153, 154, 183, 209) and PCBs (Σ_7_PCBs: 28, 52, 101, 118, 138, 153, 180). All exposures are expressed on a lipid basis in nanograms per gram lipid, measured by the creamatocrit technique described elsewhere (Mitoulas et al. 2002).

*Other variables.* We extracted information on co-variables from interviewer--administered questionnaires completed by the mothers during the third trimester of pregnancy and when their child was 14 months of age. Potential confounders assessed in the current study included maternal age, social class [professional and technical, other nonmanual, or manual occupation based on *International Standard Classification of Occupations* (International Labour Organization 1988)], education (primary, secondary, or university), and country of origin, maternal smoking during pregnancy (yes or no), parity (first child or not), child care attendance during the first 14 months of age (yes or no), duration of predominant breastfeeding [never exclusive breastfeeding, short-term (≤ 16 weeks), long-term (16–24 weeks), and very long-term (≥ 24 weeks)], and maternal consumption of fish during pregnancy (categorized according to tertiles based on a 100-item, semiquantitative food frequency questionnaire, adminis-tered at 10–13 weeks and at 28–32 weeks of pregnancy). Maternal prepregnancy body mass index (BMI), child’s gestational age, and child’s weight at birth were collected from clinical records or reported by mothers.

*Statistical methods.* Within the final study population (*n* = 290), missing values for co-variables (child care attendance, parity, maternal education, smoking during pregnancy, and duration of breastfeeding; missing for 0.35–4.5% of participants in the original cohort) were imputed by multiple imputation (Royston 2005) [see Supplemental Material, p. 4 for details (http://dx.doi.org/10.1289/ehp.1205266)]. The same method was applied to impute PBDE and OC values between 0 and the LOD or LOQ for each compound for samples that were below the LOD or the LOQ (between 0.3% and 77.6% of the samples). Because PBDE and OC concentrations were not normally distributed, we log-transformed concentrations for further analyses. Linearity of the association between log-transformed PBDEs and BSID mental and psychomotor scores was assessed using generalized additive models (GAM). Because there was no significant nonlinearity (*p* = 0.12 and *p* = 0.39 for mental and psychomotor scores, respectively; see Supplemental Material, Figure S1), log-transformed PBDE levels were modeled as continuous variables. Correlations between log-transformed PBDE and OC concentrations were assessed by performing Pearson correlations. Linear regression models were used to estimate coefficients for the association between PBDE concentrations (sum and individual congeners) and the BSID mental and psychomotor test scores. Crude models included sex, study region (Gipuzkoa or Sabadell), and mother’s region of origin (European or non-European). To determine the adjusted models, potential confounders (co-variables listed above) were included in the initial models following a forward selection procedure; variables associated with the outcome (*p* < 0.2) or that changed the β-coefficient for PBDE exposure by > 10% were included in adjusted models. Final models for both outcomes included gestational age, low birth weight (< 2,500 g), maternal social class, and maternal education, as well as sex, study region, and region of origin; models of mental score also included child care attendance and parity; models of psychomotor score also included maternal prepregnancy BMI. Confounding by PCBs, DDE, and HCB was evaluated by adding each exposure to the adjusted models (one by one and altogether) as log-transformed continuous variables. Also, we tested potential interactions between PBDEs and other POPs, and between different PBDE congeners, by modeling product terms between pairs of exposures. Exposure assessment was performed in colostrum; because breastfeeding is a source of exposure to PBDEs, and because breastfeeding has been shown to be beneficial for the neuro--psychological development of the child (Guxens et al. 2011), the interaction between exposure and breastfeeding duration (< 16 weeks vs. > 16 weeks) was also tested. Given the significantly higher PBDEs levels in the region of Gipuzkoa we also stratified analyses by region. Finally, because the children of Latin-American mothers (*n* = 17) had a different pattern of PBDE and OC exposures, we performed a sensitivity analysis excluding these children. All the analyses were done with STATA version 10 (StataCorp, College Station, TX, USA).

## Results

A description of the characteristics of the study population is shown in Table 1. PBDE concentrations were significantly higher (*p* < 0.05) in children living in Gipuzkoa, but no other maternal or child characteristics were associated to PBDE concentrations. Females, European children, those attending child care and those with primiparous mothers with a higher maternal education and social class had significantly higher average scores on the mental test. Children from mothers with a university education also had higher average psychomotor test scores (Table 1).

**Table 1:** Concentrations^*a*^ of ΣPBDEs, DDE, HCB, and ΣPCBs (ng/g lipids) and mental and psychomotor scores of the BSID test by characteristics of mothers and children (*n* = 290)

Characteristic	Exposure (GM)					BSID scores	
	Percentb	Σ7PBDEs	Σ7PCBs	HCB	DDE	Mental	Psychomotor
Child
Sex
Male	48.3	3.8	101.2	25.5	101.5	96.9	99.3
Female	51.7	4.0	113.7	26.9	108.3	100.9*	99.0
Child care attendance														
Yes	32.4	4.0	109.2	25.3	100.2	100.5	96.8
No	67.6	3.9	106.7	26.7	107.3	98.3*	100.3
Predominant breastfeeding														
Never exclusive	10.5	3.2	68.8	22.6	81.6	98.6	98.7
≤ 16 weeks	33.1	3.9	108.6*	27.1	110.8	98.1	100.6
16–24 weeks	43.0	4.2	114.0*	26.0	108.4	98.5	98.3
≥ 24 weeks	13.4	3.8	122.9*	28.0	100.6	103.2	98.6
Low birth-weight (< 2,500 g)														
Yes	2.4	2.6	118.0	28.6	90.4	93.2	93.8
No	97.6	3.9	107.2	26.2	105.3	99.2	99.3
Gestational age
< 39.8 weeks (median)	50.0	4.2	120.9	28.3	106.9	97.1	98.2
≥ 39.8 weeks (median)	50.0	3.6	95.6*	24.3	103.0	100.9	100.1
Mental BSID score
< 98.1 (median)	50.0	4.2	111.5	27.7	113.0
≥ 98.1 (median)	50.0	3.7	103.6	24.8	97.5
Psychomotor BSID score														
< 98.9 (median)	50.0	4.0	102.7	25.6	96.7
≥ 98.9 (median)	50.0	3.8	112.6	26.8	113.8
Maternal
Region of study
Gipuzkoa	30.3	5.1	183.2	33.0	142.6	99.0	98.4
Sabadell	68.5	3.5*	85.2*	23.7*	91.8*	99.2	99.5
Region of origin
Non-European	5.9	2.9	27.0	7.2	270.8	91.3	100.8
European	94.1	4.0	117.1*	28.4*	98.9*	99.5*	99.0
Age (years)
≤ 29	22.8	3.8	64.3	16.4	84.4	99.7	101.3
29–31	29.6	3.9	108.8*	26.3*	98.8	99.6	97.7
32–33	26.2	4.2	118.8*	28.7*	101.3	99.4	97.6
≥ 34	21.4	3.8	162.6*	38.6*	150.0*	96.9	100.8
Prepregnancy BMI
≤ 21.7	33.4	3.8	107.8	20.1	83.0	100.7	99.6
21.7–24	33.4	4.2	120.8	26.7*	119.4*	97.8	99.2
> 24	33.2	3.8	95.2	33.7*	116.7*	98.5	98.6
Social class
Professionals and technicians	44.5	4.1	123.0	28.3	95.4	103.0	100.4
Other nonmanual	30.0	4.0	103.0*	25.4	115.4	96.2	97.5
Manual	25.5	3.5	89.4*	23.9	110.7	95.3*	98.9
Education
Primary or without education	21.3	3.6	77.1	18.9	111.2	95.7	96.6
Secondary	45.9	3.7	105.7*	26.5*	93.3	98.1	99.3
University	32.8	4.4	136.4*	32.0*	110.7	102.4*	100.6*
Smoking during pregnancy
Yes	12.9	3.6	127.9	29.4	103.2	99.1	101.2
No	87.1	4.0	104.8	25.8	105.2	99.0	98.8
Parity
Primiparous	54.7	3.9	95.9	23.6	97.3	100.8	98.6
Multiparous	45.3	3.9	123.4*	29.8*	114.9	96.8*	99.8
Fish consumption
≤ 50.7 g/day	33.4	4.1	91.5	23.6	102.0	98.4	98.7
50.7–75.5 g/day	33.4	3.9	117.6*	29.5	101.5	99.5	98.7
> 75.5 g/day	33.2	3.7	115.5	25.9	111.7	99.1	100.0
aBecause concentrations of all compounds were not normally distributed, these were log-transformed before estimating differences in exposure between groups for each characteristic. bPercentages are presented based on imputed data. *p < 0.05 (for categorical variables, the first category is the reference).														

There were differences between included (*n* = 290) and excluded children (*n* = 1,005) [see Supplemental Material, Table S1 (http://dx.doi.org/10.1289/ehp.1205266)]. Children in the included population were less likely to attend child care during the first 14 months of life, and maternal education was lower. Included children also tended to have been breastfed for longer periods, and fewer of them were low birth weight (≤ 2,500 g). Average mental test scores were significantly higher among those included (99.2 vs. 97.0, *p* = 0.03), but average psychomotor scores were not significantly different (99.0 vs. 97.1, *p* = 0.07). There were no significant differences in average Σ_7_PBDEs, Σ_7_PCBs, HCB, or DDE exposures between included and excluded subjects.

The most frequently quantified PBDE congener in colostrum was BDE-153 (75.8%); however, BDE-209 was the congener with the highest geometric mean concentration [geometric mean (GM) = 1.03 ng/g lipid] (Table 2). DDE was the most frequently quantified OC (99.7%) and was present in higher concentrations than other individual OCs measured (GM = 104.94 ng/g lipid) (Table 2).

**Table 2:** Concentrations (ng/g lipid) of PBDEs, DDE, HCB, and PCBs in colostrum (*n* = 290).^*a*^

Compounds	< LOD (%)	LOD–LOQ (%)	> LOQ (%)	GM	Median	Minimum	Maximum
Σ7PBDEsb	NA	NA	NA	3.91	4.05	0.31	32.66
BDE-47	23.4	13.5	63.1	0.55	0.56	0.01	15.00
BDE-99	32.4	21.7	45.9	0.27	0.27	0.01	8.94
BDE-100	21.7	23.5	54.8	0.26	0.25	0.01	3.19
BDE-153	9.0	15.2	75.8	0.91	0.92	0.03	12.54
BDE-154	11.4	19.6	69.0	0.28	0.30	0.02	2.03
BDE-183	57.6	1.4	41.0	0.03	0.03	0.00	1.30
BDE-209	14.5	31.0	54.5	1.03	1.02	0.04	6.49
Σ7PCBsb	NA	NA	NA	107.50	118.60	9.17	894.45
PCB-28	34.8	16.2	49.0	1.60	1.71	0.01	16.81
PCB-52	39.0	23.1	37.9	1.66	2.13	0.00	82.51
PCB-101	77.6	17.6	4.8	1.24	1.26	0.03	22.76
PCB-118	11.7	17.2	71.1	5.68	5.76	0.38	96.68
PCB-138	0.7	1.3	98.0	23.14	26.03	1.28	222.99
PCB-153	1.0	0.7	98.3	34.50	37.15	1.24	369.01
PCB-180	1.7	0.7	97.6	30.34	32.96	1.45	266.27
HCB	2.8	1.7	95.5	26.21	28.75	0.34	251.63
DDE	0.3	0.0	99.7	104.94	98.57	2.16	8206.27
Abbreviations: LOD, number of samples below the limit of detection; LOD–LOQ, number of samples between the LOD and the LOQ; LOQ, number of samples above the limit of quantification; NA, not applicable. aValues are provided after multiple imputation of those which were below LOD or LOQ. bOnly those congeners detected in > 20% of the samples are shown and included in the final sum.														

Most of the PBDE congeners were moderately or highly correlated (*r* between 0.30 and 0.90), except for correlations of BDE-153 and BDE-183 with BDE-47, BDE-99, and BDE-100 (*r* between 0.05 and 0.24) [see Supplemental Material, Table S2 (http://dx.doi.org/10.1289/ehp.1205266)]. DDE was not correlated with any of the PBDEs (*r* between –0.02 and 0.07). Correlations between HCB or PCBs and BDEs 100, 153, 154, 183, and 209 ranged from 0.16 and 0.63, whereas correlations with BDEs 47 and 99 were lower (*r* between 0.03 and 0.14).

Σ_7_PBDE concentrations in colostrum were negatively, but not statistically significantly associated with the mental test score after adjusting for potential confounding variables (adjusted β per log ng/g lipid = –2.25; 95% CI: –4.75, 0.26) (Table 3). This association was weaker after also adjusting for Σ_7_PCBs, HCB, and DDE (β = –1.85; 95% CI: –4.76, 1.06). There was little evidence for an association with psychomotor score either before (β = –1.39; 95% CI: –3.78, 0.99) or after adjustment for OCs. Analyses of individual PBDE congeners showed similar results, except for BDE-209, which was statistically significantly associated with mental test scores (β = –2.40; 95% CI: –4.79, –0.01) (Table 3). After adjusting for Σ_7_PCBs, HCB, and DDE, the coefficient was reduced somewhat and was no longer significant (β = –2.10; 95% CI: –4.66, 0.46).

**Table 3:** Regression analyses results (crude, adjusted, and OCs adjusted) for the total population (*n* = 290).

Compounds and models	Mental score		Psychomotor score	
	βa (95% CI)	p-value	βa (95% CI)	p-value
Σ7PBDEs										
Crude modelb	–2.33 (–4.90, 0.23)	0.07	–1.32 (–3.66, 1.02)	0.27
Adjusted modelc	–2.25 (–4.75, 0.26)	0.08	–1.39 (–3.78, 0.99)	0.25
Multipollutant modeld	–1.85 (–4.76, 1.06)	0.21	–1.48 (–4.23, 1.27)	0.29
PBDE congeners (adjusted)c										
BDE-47	–1.22 (–2.89, 0.46)	0.15	–0.55 (–2.16, 1.05)	0.50
BDE-99	–1.37 (–2.99, 0.25)	0.10	0.13 (–1.44, 1.69)	0.87
BDE-100	–1.36 (–3.48, 0.77)	0.21	0.10 (–1.92, 2.11)	0.93
BDE-153	–1.29 (–3.55, 0.96)	0.26	–1.05 (–3.18, 1.07)	0.33
BDE-154	–0.97 (–3.37, 1.42)	0.42	–0.60 (–2.86, 1.67)	0.60
BDE-183	–0.34 (–1.24, 0.56)	0.46	–0.02 (–0.89, 0.86)	0.97
BDE-209	–2.40 (–4.79,-0.01)	0.05	–1.48 (–3.74, 0.79)	0.20
BDE-209
Multipollutant modeld	–2.10 (–4.66, 0.46)	0.11	–1.26 (–3.67, 1.15)	0.31
aβ coefficient per unit of log ng/g lipid. bCrude models already adjusted for region of study, sex and region of origin of the mother. cMental score model adjusted for region of study, sex, region of origin of the mother, gestational age, low birth weight, maternal social class and education, child care attendance and parity. Psychomotor score model adjusted for region of study, sex, region of origin of the mother, gestational age, low birth weight, maternal social class and education, and maternal prepregnancy BMI. dMultipollutant models include those variables indicated in crude models plus Σ7PCBs, HCB, and DDE.										

We found no statistically significant interactions between different compounds (interaction *p*-values ranged from 0.12 to 0.87) (data not shown). Associations between PBDEs and mental or psychomotor test scores did not differ significantly by breastfeeding duration (interaction *p*-values for mental test score: Σ_7_PBDE *p* = 0.42, BDE-209 *p* = 0.25; for psychomotor test score: Σ_7_PBDE *p* = 0.14, BDE-209 *p* = 0.35). However, the association between BDE-209 and the mental test score was –1.07 (95% CI: –4.85, 2.71) in children breastfed for ≤ 16 weeks (*n* = 126) compared with –3.48 (95% CI: –6.85, –0.12) in children breastfed for > 16 weeks (*n* = 164). Excluding 17 children whose mothers were born in Latin America did not change results substantially. Results were similar for children from Gipuzkoa and Sabadell (data not shown).

## Discussion

Results suggest an association between increasing PBDE concentrations in colostrum, particularly BDE-209, and a worse cognitive development in the first year of life. There was little evidence of an association with psychomotor development. After adjusting for PCBs, DDE, and HCB, the association between BDE-209 and cognitive development remained, but was slightly weaker.

*Levels of exposure and congeners.* In previous studies PBDEs were measured in maternal or cord blood (Gascon et al. 2011; Herbstman et al. 2010; Roze et al. 2009; Shy et al. 2011; Vizcaino et al. 2011), which are matrices where the capacity to detect PBDEs is lower than in breast milk or placenta because of their lower fat content (Antignac et al. 2009; Gomara et al. 2007). Thus, it is hard to compare levels of the present study with those of previous studies. Only one study of 70 children in Taiwan that measured PBDEs in breast milk detected all congeners in almost 100% of the samples (Chao et al. 2011), whereas in our study the number of samples above the LOD ranged between 22.4% and 91.0%. Comparing levels among the different studies available, these were similar between studies, except those of BDE-154 and BDE-209, which were higher in the present study population, and BDE-183, lower than in the Taiwanese study (Chao et al. 2011) but detected in more samples than in the U.S. study (Herbstman et al. 2010). Because BDE-209 has a relatively short half-life [an occupational study calculated it to be 15 days in serum (Thuresson et al. 2006)], the presence of higher concentrations of this congener in breast milk samples indicates recent exposure of the mothers of our study population, unless continuous exposure sources and habits are assumed. However, further research is needed to improve our very limited knowledge of exposure determinants of different PBDE congeners (Antignac et al. 2009; Frederiksen et al. 2010a; Gomara et al. 2007; Herbstman et al. 2007; Shy et al. 2011; Thomsen et al. 2010; Vizcaino et al. 2011).

*Association between PBDEs exposure and neuro-psychological development.* Only a few small birth cohort studies (between 36 and 118 subjects) have studied associations between PBDEs and children’s neuro-psychological development (Chao et al. 2011; Gascon et al. 2011; Herbstman et al. 2010; Roze et al. 2009; Shy et al. 2011). Exposure levels, analysis methods, confounders, the neuro-psychological development test used, and the age at testing have all varied among these studies, which may explain why results have differed. Two European studies (Gascon et al. 2011, *n* = 88; Roze et al. 2009, *n* = 62, respectively) reported no significant associations between prenatal PBDEs exposure and children’s neuro-psychological development or behavior at ages between 4 and 6 years of age (Gascon et al. 2011; Roze et al. 2009). A U.S. study with higher levels of exposure than the European studies reported that prenatal PBDE levels were negatively associated with neuro-psychological test scores at ages between 1 and 6 years (*n* = 96–118), although results were not statistically significant at all ages or for all PBDE congeners (Herbstman et al. 2010). For example, at age 1 year no significant associations between PBDE congeners such as BDEs 47, 99, 100, or 153 and mental scores of the BSID-II were found (Herbstman et al. 2010). In two small Taiwanese birth cohort studies (*n* = 70 and *n* = 36), with low PBDE levels of exposure, only a subset of the multiple congeners analyzed were associated with neuro-psychological test scores around the first year of age (Chao et al. 2011; Shy et al. 2011). Chao et al. analyzed 14 PBDE congeners in breast milk: Increasing concentrations of BDE-196 were associated with better language skills, whereas increasing concentrations of BDE-209 were associated with worse BSID test scores at 8–12 months of age (Chao et al. 2011).

We did not measure BDE-196 for the present study, but our results for BDE-209 are consistent with those reported by Chao et al. (2011). We found no significant associations with psychomotor test score. The two Taiwanese studies reported no significant associations with motor skills (Chao et al. 2011; Shy et al. 2011), whereas the U.S. study reported decreased BSID-II psycho-motor scores (at age 1 year) in association with increased BDE-47 (Herbstman et al. 2010).

Our study, which provides some evidence for a negative effect of PBDE exposure on early cognitive development, is larger than previous studies, and we found consistent negative associations with BSID mental scale scores across all of the PBDE congeners analyzed, though associations were not statistically significant. The BSID is one of the most widely used tools available in Spanish to assess neuro-development at such young ages; however, the BSID has sometimes shown a low predictive value for later performance on general cognition and intelligence tests (Bayley 1977); therefore, the associations reported here need to be followed up at older ages.

*Mechanisms.* In the present study BDE-209 was present in higher concentrations in colostrum than the other congeners evaluated, which might explain why we found a negative association between BDE-209 and a measure of cognitive development. BDE-209 belongs to the last type of PBDE (decaPBDEs) to be banned in electronic equipment in Europe (in 2008) and is scheduled to be banned in the United States by 2013 (Bromine Science and Environmental Forum 2011). In *in vitro* studies, it has been shown to induce oxidative stress and apoptosis, as well as other cellular effects (Costa and Giordano 2011), but its toxicity is lower than that of other PBDE congeners, and it has a shorter half-life (15 days in adult’s serum compared with other PBDE congeners whose half-lives can range from 18 to 94 days), and does not readily enter cells because of its bulky configuration (Costa and Giordano 2011). In fact, its transfer from maternal serum to fetus seems to be quite limited compared with other congeners, according to a study that implemented a human placenta perfusion system (Frederiksen et al. 2010b). Thus, the association found for BDE-209, if causal, could be the result of its bioaccumulated metabolites (e.g., BDEs 203 and 206), which are more toxic and more stable, and cross the placenta and reach the brain more easily than BDE-209 (Costa and Giordano 2011; Frederiksen et al. 2010b). The more toxic hydroxylated PBDE metabolites (OH-PBDEs) could also be the responsible compounds (Dingemans et al. 2008). In any case, further and larger studies are need to confirm the present results and to rule out chance, bias, or potential confounding that may not have been taken into account in the present study.

*The influence of other OCs.* It is known that routes of exposure can differ between PBDE congeners and also between PBDEs and other POPs (Fischer et al. 2006; Johnson et al. 2010; Liberda et al. 2011; Trudel et al. 2011; Watkins et al. 2012). This may explain why some of the PBDE congeners were more highly correlated with PCBs or HCB than others in the present study. BDE-209 was moderately correlated with PCBs and HCB; this could explain why the association between BDE-209 and neuro-psychological scores became somewhat weaker after adjusting for other POPs. Thus, confounding by other POPs cannot be ruled out. In a previous birth cohort study conducted in Menorca, Spain, evidence of confounding by other OCs was not found, perhaps because the correlation between postnatal BDE-47 (the only congener assessed in the previous study) and the different OCs was very low (Gascon et al. 2011). In the present study the correlation between BDE-47 and OCs also was very low, whereas the correlation between Σ_7_PBDE or BDE-209 and the OCs was somewhat higher. These results indicate that further and bigger studies on the effects of PBDEs should also include other POPs in the analyses in order to understand better the role of each compound.

Regarding synergistic effects of co-exposures to different compounds, in the present study we did not observe significant interaction effects (*p* < 0.05) between PBDEs and other POPs, between BDE-209 and other PBDE congeners, or between BDE-47 and PCB-153. Synergistic effects of BDE-47 and PCB-153 have been reported in animal and *in vitro* models (He et al. 2009b, 2011; Pellacani et al. 2012). However, we had limited power to test interactions in our small study population. Further, we tested inter-actions only between pairs of exposures, but these co-exposures are in fact multiple and diverse within individuals; only larger studies may be able to clarify this issue.

*Strengths and limitations of the study design.* In the present study we chose colostrum to measure exposure to PBDEs because of its higher fat content, which facilitates PBDEs detection. Nonetheless, different PBDE congeners have different transfer rates from maternal serum to cord blood, placenta, or breast milk. Unfortunately, there are no exact data on the transfer rates for each congener and biological matrix (Antignac et al. 2009; Frederiksen et al. 2010a, 2010b; Gomara et al. 2007; Vizcaino et al. 2011). We cannot therefore ensure that levels observed in breast milk in the present study are a total reflection of prenatal exposure. Additionally, postnatal exposure is determined not only by the levels measured in colostrum, but also by the breastfeeding duration of the child. Thus, in case BDE-209 is actually causing the effects found in the present study, we cannot determine whether pre- or postnatal exposure or a combination of both exposures may have been responsible for the observed association.

In the present study we found no significant interaction between exposure and breastfeeding duration, but in the group of children breastfed for a longer period the association between BDE-209 exposure and neuro-development impairment was somewhat stronger. These results could indicate effects of postnatal exposure, but they must be interpreted with caution because our stratified groups were small. Further, associations in the longer breastfeeding group may be underestimated because the higher social class and education level of these mothers may provide a more advantageous environment for neuro-development. Because up to now beneficial effects of breastfeeding have been considered more important than the potential deleterious effects of POPs (Ribas-Fito et al. 2001, 2007a), the effects of postnatal exposure should be explored further in children both at young and older ages in studies with larger study populations.

There were significant differences between included and excluded children, which could have lead to selection bias. The most important difference was the average BSID mental test score, which was significantly higher among the included children. This may reflect the smaller proportion of children with low birth weight (< 2,500 g), which is a known risk factor for lower neuro-psychological development (Pyhala et al. 2011). However, we do not think this would bias the associations observed in the children included in the present study.

## Conclusion

Our findings suggest an association between PBDE concentrations in colostrum and impaired infant cognitive development, particularly for BDE-209, which adds to existing evidence that supports the banning of these chemical compounds. Future studies should include larger study populations and a longer follow-up to assess the potential long-term effects of PBDEs and its metabolites. Inclusion of other POPs in the analyses is also recommended given their similar characteristics to PBDEs. Further research should also try to detangle the role of BDE-209 metabolites and OH-PBDEs congeners, given their higher toxicity and capacity to reach brain tissue, as well as potential synergistic effects of different compounds.

## Supplemental Material

(1.2 MB) PDF
