# Supplementary material for: Polybrominated Diphenyl Ethers (PBDEs) in Breast Milk and Neuropsychological Development in Infants
Source: Environ Health Perspect. 2012 Sep 25;120(12):1760–5. doi: 10.1289/ehp.1205266 (PMC3548276; doi:10.1289/ehp.1205266)
Supplement: (1.2 MB) PDF [file ehp.1205266.s001.pdf]

SUPPLEMENTAL MATERIAL

Polybrominated Diphenyl Ethers (PBDEs) in Breast Milk and Neuropsychological Development in Infants

Mireia Gascon, Marta Fort, David Martínez, Anne-Elie Carsin, Joan Forns, Joan O. Grimalt, Loreto Santa Marina, Nerea Lertxundi, Jordi Sunyer, Martine Vrijheid.

TABLE OF CONTENTS

Liquid-liquid extraction protocol and LOD and LOQ determination……………………………..…………..….2

Description of the imputation procedure…………………………………………………………..……………...4

Figure S1. Generalized additive models (GAM)……………………………………..………………………5

Table S1. Excluded and included participants: characteristics and differences……………………..……...6

Table S2. Correlation between the different POPs measured in colostrum samples…………………………7

Exposure assessment: liquid-liquid extraction protocol to measure POPs in colostrum and description of the limitis of detection (LOD) and quantification (LOQ).

Colostrum was collected the first 48 to 96 hours' postpartum at the hospital by an experienced nurse. The sample was collected in the morning at the end of the feeding, in sterile polypropylene tubes, by mechanical expression of one breast using a breast pump. Milk was transported to the laboratory in ice boxes less than 2 hours after collection, where samples were stored at -80℃ until analysis. OCs (DDE, HCB and P CB congeners 28, 52, 101, 118, 138, 153, 180) and PBDEs (congeners 17, 28, 47, 66, 71, 85, 99, 100, 138, 153, 154, 183, 190, 209) were extracted following a liquid-liquid extraction protocol. Colostrum (1 mL), n-hexane (3 mL) and concentrated H_2SO_4 (3 mL) were added in 10 mL centrifuge glass tubes. The mixture, spiked with 2-ethylhexyl-2,3,4,5-tetrabromobenzoate (TBB) and PCB 209 as surrogate standards, was mixed (vortex, ca. 1500 rpm, 30 s) and centrifuged (ca. 3500 rpm, 10 m). Supernatant was separated, and 2 mL of hexane were added to the resting acid fraction, mixed in vortex, centrifuged twice, and yielded together with the first n–hexane fraction. Concentrated H_2SO_4 (3mL) was added to the final volume of 7 mL of extract, mixed with vortex (ca. 1500, 90 s) and centrifuged (ca. 3500, 20 m). Clean extract was then evaporated under a gentle N_2 stream to near dryness and dissolved with isooctane to a glass chromatographic vial. Prior to injection, the extract was evaporated again and 100 µL of PCB142 were added as an internal standard for OCs. The extract (2 µL) was injected automatically in a gas chromatograph with electron capture detection (GC/ECD model 6890, Agilent; Palo Alto, CA) in split-spitless mode, with a 60m DB-5 column (J&W/Agilent) using helium as carrier gas (1.5 mL/min). The temperature program was 90℃ for 2 min, 15 ℃/min to 130℃, and finally 4 ℃/min to 290℃, held for 15 min. Quantification was performed using PCB142 as an internal standard to correct for volume. Results were corrected with recoveries of TBB and PCB209 (70-85%). After OCs were analyzed, samples were re-evaporated again and BDE 118 (30 µL) and [^{13}C]-BDE 209 (20 µL) were added as internal standards for PBDE analysis. The extract (1 µL) was injected in an Agilent 6890N GC coupled to a 5975 mass spectrometer (Agilent Technologies, Palo Alto, CA, USA) operating in negative chemical ionization mode (NICI), in split/splitless mode, with a 15 m DB-5MS column (J&W/Agilent) using helium as carrier gas (1.5 mL). The temperature program

was 90℃ for 1.5 min, 40℃/min to 200℃, 5℃/min to 275℃, 40℃/min to 300℃, held for 10 minutes, and 10℃/min to 310℃, held for 2 minutes. Quantification was performed using BDE118 for most PBDE congeners and [^{13}C]-BDE209 for BDE209 as internal standards to correct for volume, and results were corrected with recoveries of PCB209. This method performed satisfactorily in repeated international intercalibration exercises within the AMAP Ring Test Proficiency Program for POPs (Centre de Toxicologie Institut National de Santé Publique du Québec, Québec, Canada). For both OCs and PBDEs, limits of detection (LOD) and quantification (LOQ) were calculated from blanks (i.e., the standard deviation times three LOD or five LOQ). The limits of detection (LOD) for PBDE ranged from 0.0006 ng/ml (BDE17) to 0.019 ng/ml (BDE209), whereas the limit of quantification (LOQ) ranged from 0.001 ng/ml (BDE17) to 0.03 ng/ml (BDE209). For OCs, the LOD ranged from 0.004 ng/ml (PCB28) to 0.08 ng/ml (HCB), and the LOQ ranged from 0.01 ng/ml (PCB28) to 0.10 ng/ml (PCB118).

Description of the imputation procedure.

- **Software used and key setting:** STATA 10.1 software (Stata Corporation, College Station, Texas) –*ice* command (with 20 cycles).

- **Number of imputed datasets created:** 20.

- **Variables included in the imputation procedure:**

  - **Child variables**: gestational age, duration of predominant breastfeeding, birth weight and sex and day care attendance.

  - **Maternal variables**: age, BMI, smoking, social class and education, fish consumption, parity, thyroid hormone levels (TSH, FT4 and TT3), country of origin and levels of lipids and the different POPs measured in colostrum [HCB, β-HCH, PCBs (28, 52, 101, 118, 138, 153, 180), DDT, DDE, DDD and BDEs (47, 99, 100, 153, 154, 183, 209)] and in maternal serum [PCB(138,153,180), DDE, β-HCH].

  All samples were included as continuous variables except the following:

  - **Dichotomous variables:** sex, day care attendance, maternal smoking.

  - **Categorical variables (all categories available included):** maternal social class (6 categories) and education (3 categories), parity (3 categories), country of origin (11 categories).

- **Treatment of non-normally distributed variables:** log-transformed (POPs).

- **Treatment of binary/categorical variables:** logistic, ordinal, and multinomial models.

- **Statistical interactions included in imputation models:** imputations were done separately by each region (Sabadell and Gipuzkoa).

Figure S1. Generalized additive models (GAM) for the associations between log Σ_7PBDEs and mental and psychomotor scores.

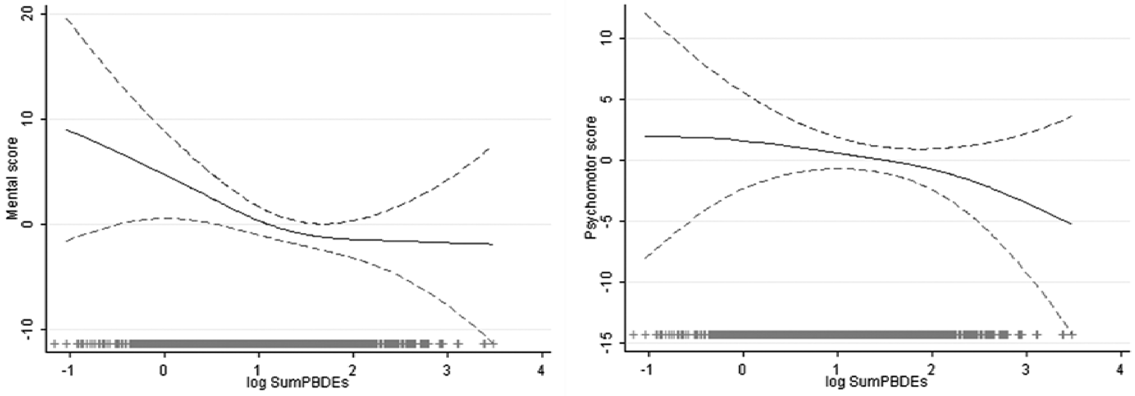

Mental score model adjusted for: region of study, sex, region of origin of the mother, gestational age, low birth weight, maternal social class and studies, day-care attendance and parity (p for non-linearity=0.12). Psychomotor score model adjusted for: region of study, sex, region of origin of the mother, gestational age, low birth weight, maternal social class and studies and maternal pre-pregnancy BMI (p for non-linearity=0.39).

Table S1. Characteristics and differences between the study population (N=290) and those not included (N=1005).

Characteristics	Excluded (N=1005)		Included (N=290)		
	N	%, Mean ± SD, or GM (min-max)	N	%, Mean ± SD, or GM (min-max)	p-value
**Child**					
Sex					
Female	460	45.8	150	51.7	0.42
Male	479	47.7	140	48.3	
missing	66	6.6	0	0.0	
Day care attendance					
No	452	45.0	191	65.9	0.003
Yes	324	32.2	89	30.7	
missing	229	22.8	10	3.5	
Predominant breastfeeding					
Never exclusive	164	16.3	30	10.3	<0.001
≤ 16 weeks	247	24.6	91	31.4	
16-24 weeks	267	26.6	119	41.0	
≥24 weeks	78	7.7	37	12.8	
missing	249	24.8	13	4.5	
Low birth-weight (≤2500g)					
Yes	50	5.0	7	2.4	0.04
No	878	87.4	283	97.6	
missing	77	7.6	0	0.0	
Gestational age (weeks)	940	39.7 ± 1.6	290	39.8 ± 1.2	0.29
BSID score					
Mental	817	97.0 ± 15.7	290	99.2 ± 13.3	0.03
Psychomotor	817	97.1 ± 15.8	290	99.0 ± 14.9	0.07
**Maternal**					
Region of study					
Gipuzkoa	550	54.7	88	30.3	<0.001
Sabadell	455	45.3	202	69.7	
Region of origin					
Non-European	33	3.3	17	5.9	0.07
European	925	92.0	273	94.1	
missing	47	4.7	0	0.0	
Maternal age (years)	816	32.2 ± 3.9	290	31.8 ± 4.2	0.12
Pre-pregnancy BMI	1004	23.3 ± 4.2	290	23.4 ± 3.8	0.69
Social Class					
Professionals & technicians	482	48.0	129	44.5	0.17
Other non manual	318	31.6	87	30.0	
Manual	205	20.4	74	25.5	
Education					
Primary or without education	214	21.3	61	21.0	0.03
Secondary	379	37.7	132	45.5	
University	408	40.6	95	32.8	
missing	4	0.4	2	0.7	

Table S1 (cont.)

Characteristics	Excluded (N=1005)		Included (N=290)		
	N		N		p-value
Smoking during pregnancy					
No	791	78.7	250	86.2	0.50
Yes	134	13.3	37	12.8	
missing	80	8.0	3	1.0	
Parity					
Primiparous	483	48.1	158	54.5	0.18
Multiparous	333	33.1	131	45.2	
missing	189	18.8	1	0.3	
Fish intake during pregnancy (g/week)	1004	65.8 ± 29.8	290	68.7 ± 33.1	0.16
Organochlorine compounds (ng/g lipid)[a]					
Σ_7PBDEs[b]	58	3.3 (1.03-13.11)	290	3.50 (0.84-29.53)	0.56
Σ_7PBCs[c]	58	117.2 (11.53-664.66)	290	106.67 (9.50-704.66)	0.44
HCB	58	27.0 (1.48-145.84)	290	25.75 (1.13-251.63)	0.75
DDE	58	118.4 (9.99-3255.90)	290	104.71 (1.34-8206.27)	0.42

PBDEs= polybrominated diphenyl ethers, DDE= dichlorodiphenyldichloroethylene, HCB= hexachlorobenzene, PCBs= polychlorinated biphenyls, p-value for the differences of each characteristic between included and excluded.

[a]As concentrations of all compounds were not normally distributed, these were log-transformed before calculating differences of exposure between groups of each characteristic.

[b]Σ_7PBDEs: 47, 99, 100, 153, 154, 183, 209.

[c]Σ_7PCBs: 28, 52, 101, 118, 138, 153, 180.

Table S2. Correlation between the different POPs measured in colostrum samples (N=290).

**Compounds**	**Σ_7PBDEs**	**BDE47**	**BDE99**	**BDE100**	**BDE153**	**BDE154**	**BDE183**	**BDE209**	**Σ_7PCBs[a]**	**HCB**	**DDE**
**Σ_7PBDEs**	1										
BDE47	0.82*	1									
BDE99	0.78*	0.90*	1								
BDE100	0.81*	0.85*	0.84*	1							
BDE153	0.62*	0.18*	0.12*	0.24*	1						
BDE154	0.63*	0.34*	0.31*	0.45*	0.52*	1					
BDE183	0.30*	0.08	0.05	0.10	0.32*	0.33*	1				
BDE209	0.67*	0.38*	0.36*	0.38*	0.32*	0.45*	0.23*	1			
**Σ_7PCBs[a]**	0.38*	0.11	0.03	0.16*	0.49*	0.64*	0.28*	0.33*	1		
**HCB**	0.31*	0.14*	0.14*	0.22*	0.28*	0.49*	0.17*	0.23*	0.63*	1	
**DDE**	0.02	-0.01	-0.02	0.04	0.02	0.07	0.07	0.03	0.08	0.13*	1

[a]Σ_7PCBs: 28, 52, 101, 118, 138, 153, 180.
Spearman correlation *$p<0.05$.
